# Effect of cancer cachexia on first-line chemotherapy in patients with advanced pancreatic cancer: a claims database study in Japan

**DOI:** 10.1007/s10147-024-02467-6

**Published:** 2024-02-14

**Authors:** Junji Furuse, Fumihiko Osugi, Koji Machii, Koji Niibe, Toshimitsu Endo

**Affiliations:** 1 Department of Gastroenterology, Kanagawa Cancer Center, 2-3-2 Nakao, Asahi-Ku, Yokohama, 241-8515 Japan; 2 Medical Affairs, Ono Pharmaceutical Co., Ltd., 8-2, Kyutaromachi 1-Chome Chuo-Ku, Osaka-Shi, 541-8564 Japan; 3 Digital Strategy & Planning, Ono Pharmaceutical Co., Ltd., 8-2, Kyutaromachi 1-Chome Chuo-Ku, Osaka-Shi, 541-8564 Japan

**Keywords:** Cancer cachexia, Pancreatic cancer, Database study, Time-to-treatment failure, Number of doses

## Abstract

**Background:**

Cancer cachexia is a multifactorial syndrome leading to progressive functional impairment. How cachexia affects the treatment course of chemotherapy in patients with pancreatic cancer has not been well understood.

**Methods:**

This is an exploratory, retrospective, observational cohort study using the Japanese medical claims database from Medical Data Vision Co., Ltd. The study population included patients diagnosed with pancreatic cancer in whom first-line FOLFIRINOX (FFX) or gemcitabine plus nab-paclitaxel (GnP) was initiated between October 1, 2018, and September 30, 2020. In this study, we defined patients with cancer cachexia as those who had a weight loss of ≥ 5% in the preceding 6 months. The primary outcome was time-to-treatment failure (TTF). The observation period was six months from the initiation of first-line FFX or GnP treatment.

**Results:**

A total of 1897 patients (421 patients into the cachexia group; 1476 patients into the non-cachexia group) were analyzed in this study. The median TTF was 121 days (95% confidence interval [CI] 94–146) in the cachexia group and 143 days (95% CI 134–152) in the non-cachexia group. The hazard ratio for TTF of the cachexia versus non-cachexia group was 1.136 (95% CI 0.979–1.319). The median number of doses was two doses fewer in the cachexia group than in the non-cachexia group for both FFX and GnP.

**Conclusion:**

Cancer cachexia was suggested to be associated with shorter TTF and a reduced number of doses in patients with pancreatic cancer who received first-line FFX or GnP treatment.

Clinical Trial Registration clinicaltrials.jp: UMIN000045820.

**Supplementary Information:**

The online version contains supplementary material available at 10.1007/s10147-024-02467-6.

## Introduction

Pancreatic cancer is one of the most common cancers worldwide, with the incidence predicted to reach 18.6 per 100,000 by 2050 [1]. Although the overall outcome of pancreatic cancer has improved in the past decade, only about 6% of patients with pancreatic cancer will live for five years [2]. For patients with metastatic pancreatic cancer, FOLFIRINOX (FFX; a combination of fluorouracil, leucovorin, irinotecan, and oxaliplatin) and gemcitabine plus nab-paclitaxel (GnP) have been used globally [3, 4]. The FFX and GnP regimens also demonstrated clinical benefit for chemotherapy‑naïve Japanese patients with metastatic pancreatic cancer in phase II trials [5, 6], and have consequently become the standard treatment for patients with metastatic pancreatic cancer in Japan. Additionally, a modified FFX regimen for first-line treatment has been developed for Japanese patients with pancreatic cancer [7, 8].

Cancer cachexia is a multifactorial syndrome characterized by anorexia and ongoing weight loss. It is observed in 50–80% of patients with cancer [9] and its prevalence is especially high in pancreatic cancer (> 60%) [9–12]. One of the diagnostic criteria for cachexia is ≥ 5% weight loss in six months [9]. Cachexia also affects the clinical outcome in patients with cancer; weight loss prior to chemotherapy treatment is associated with increased toxicities from chemotherapy, which can lead to dose reduction or discontinuation of treatment, and with shorter survival [13]. Several studies have reported the association between cachexia and poor prognosis in advanced pancreatic cancer [14–18]; however, these studies included only a small number of Japanese patients. In order to gain a comprehensive understanding of how cachexia affects the treatment course of advanced pancreatic cancer in the Japanese clinical setting, evaluation on a large scale with real-world dataset is required.

Studies using large real-world datasets have been attracting a high level of interest globally [19]. Medical Data Vision (MDV) is a large medical claims database comprising the clinical data of more than 40 million patients (as of February 2023) recorded in diagnosis procedure combination hospitals in Japan, with information on diagnosis, drug prescription, medical procedures, and hospitalization [19]. In this retrospective observational cohort study, we screened patients with pancreatic cancer who had ≥ 5% weight loss in the preceding six months (one of the diagnostic criteria of cachexia) by using the weight data in the MDV database, and compared the treatment course and clinical outcomes of patients with and without cachexia. Here, we report the primary results of the study, focusing on the association between cachexia and treatment course of first-line chemotherapy (FFX or GnP) in Japanese patients with pancreatic cancer.

## Patients and methods

### Study design

This is an exploratory study with the use of anonymized and deidentified datasets from the MDV database (Medical Data Vision Co, Ltd., Tokyo, Japan). This study was not subject to the Japanese Ethical Guidelines for Medical and Health Research Involving Human Subjects and therefore did not require a review by an ethical committee. This study was registered on the University Hospital Medical Information Network-Clinical Trials Registry (UMIN000045820).

### Study population

We included patients with pancreatic cancer who received chemotherapy in the first-line setting. The inclusion criteria were: a definitive diagnosis of invasive ductal carcinoma of the pancreas according to Japanese disease codes corresponding to International Statistical Classification of Diseases and Related Health Problems 10th edition (ICD-10) code C25 (those who had pancreatic malignancies other than invasive ductal carcinoma were excluded); FFX or GnP regimen as first-line chemotherapy started at age ≥ 20 years and between October 1, 2018, and September 30, 2020; body weight data at the initiation of first-line chemotherapy; and body weight data recorded at least twice within six months prior to the initiation of the first-line chemotherapy. The exclusion criteria were: pancreatic cancer stage < 3 on the first day of first-line chemotherapy; initial chemotherapy for < 60 days followed by pancreatic surgery (defined by Japan-specific procedure codes); surgery for pancreatic cancer between the most recent diagnosis of pancreatic cancer and the initiation of first-line chemotherapy; relapse without surgery during initial cancer treatment; relapse without initial cancer information; relapse without postoperative chemotherapy during initial cancer treatment; relapse < 6 months after completion of initial postoperative chemotherapy; those who had pancreatic cancer stage ≥ 3 at the first onset, received preoperative chemotherapy for 60 days or longer during the initial cancer treatment, and subsequently had relapse; no record of activities of daily living (ADL) or cancer stage data at the initiation of first-line chemotherapy; and irregular chemotherapy regimen after the initiation of first-line chemotherapy.

### Definition of cancer cachexia

**Fig.1:**
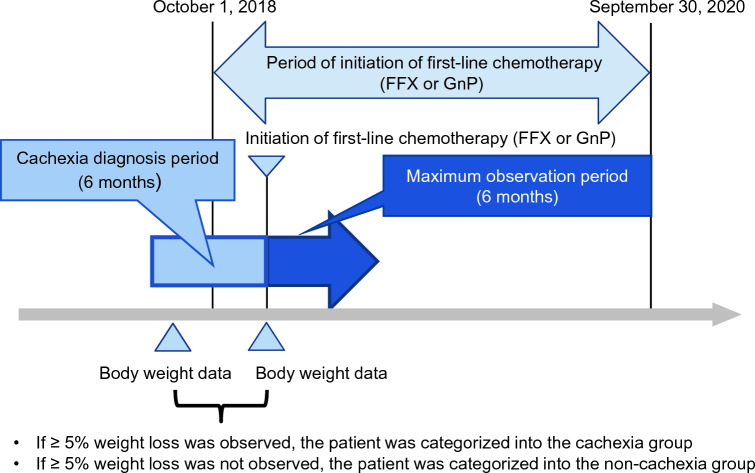
Study design. FFX, FOLFIRINOX (fluorouracil + leucovorin + irinotecan + oxaliplatin); GnP, gemcitabine + nab-paclitaxel

Following one of the criteria for diagnosing cachexia [9], we defined patients with cachexia as those who had a weight loss of ≥ 5% when the baseline weight was compared to the maximum weight (Figure 1). We used weight data recorded at the initiation of first-line chemotherapy as the baseline weight. If weight data existed both before and after the initiation of first-line chemotherapy, that recorded before the initiation of chemotherapy was used as the baseline weight. If more than two weight data existed before or after the initiation of the first-line chemotherapy, that recorded on the date closest to the initiation of chemotherapy was used as the baseline weight. The maximum weight was defined as the maximum weight recorded between six months before the initiation of first-line chemotherapy and one day before the recording of baseline weight.

### Outcomes

The maximum observational period was six months after the initiation of first-line chemotherapy. The primary outcome was time-to-treatment failure (TTF), which was defined as the interval between treatment initiation and premature discontinuation of the regimen. For TTF analysis, if patients received a drug that was not included in the regimen they followed, or if there was no prescription of chemotherapy drugs for 30 days or more (i.e., 30 days or more passed after the last chemotherapy drug prescription), the last chemotherapy drug prescription date was recorded as an event. Other outcomes included overall survival (OS), number of doses, relative dose intensity (RDI), and changes in body weight from baseline after the initiation of first-line chemotherapy. The OS was defined as the interval between the initiation of first-line chemotherapy and death from any cause during hospitalization. The number of doses was defined as the number of prescriptions applied during TTF. A prescription was counted as one dose regardless of any reduction in the number of drugs in the regimen. For FFX, three days of prescription was counted as one dose. We defined RDI as the amount of prescribed drug divided by the full dose of the drug in the standard regimen. Because the claims data only included the number of units ordered and not the actual amount administered to the patient, we used the prescribed amount of the drug for the calculation. The full dose of the drug for a patient was estimated based on the patient’s height and weight data recorded at the initiation of the chemotherapy (baseline) or if height and weight data existed after the baseline, that recorded on the date close to the prescription date, placing a priority on the date before the prescription date. Regarding FFX, conventional FFX was used in the calculation of RDI.

### Statistical analysis

For TTF and OS, the Kaplan–Meier method was used to estimate medians and their corresponding 95% confidence intervals (CIs), and the difference between cachexia and non-cachexia groups was compared by the log-rank test. For TTF, if patients underwent surgery, they were censored on the last prescription date prior to the surgery. Patients who were followed for less than 30 days from the last chemotherapy drug prescription were also censored on the last prescription date. For OS, if patients underwent surgery, they were censored on the date of the surgery, and if the date of their death was not recorded, they were censored on the day on which the last record exists.

For multivariable analysis, the Cox regression analysis was used for TTF and linear regression analysis was used for the number of doses and RDI. For TTF, hazard ratio (HR) and 95% CI were calculated for the Cox regression analysis. For the number of doses, the beta coefficient, 95% CI, and *P*-value were calculated with generalized linear model. For RDI, difference, 95% CI, and *P*-value were calculated with linear regression analysis.

All statistical tests were two-sided with a significance level of 0.05. SAS® version 9.4 (SAS Institute Inc., Cary, NC) and R (version 4.0.2) were used to conduct the analysis.

## Results

### Patient selection

**Fig.2:**
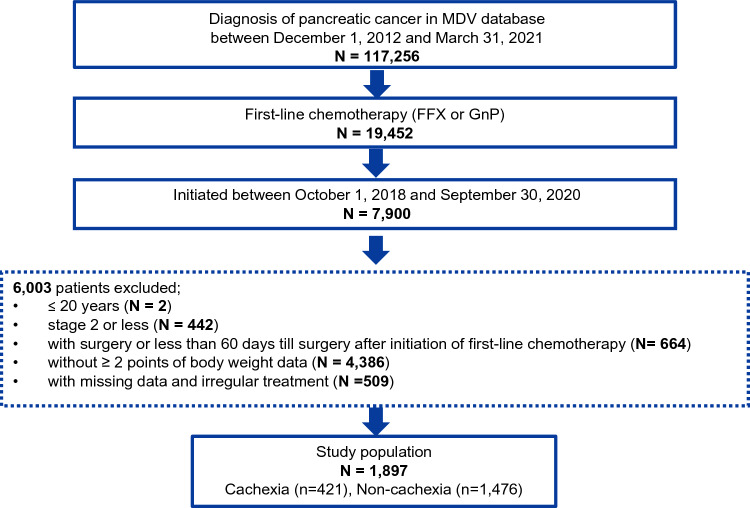
Flowchart of the study cohort. FFX, FOLFIRINOX (fluorouracil + leucovorin + irinotecan + oxaliplatin); GnP, gemcitabine + nab-paclitaxel

A total of 117,256 patients with a diagnosis of pancreatic cancer were recorded in the MDV database between December 1, 2012, and March 31, 2021 (Figure 2). Of those, 7900 patients started first-line FFX or GnP treatment between October 1, 2018, and September 30, 2020. After excluding the patients who did not meet the other inclusion criteria, 1897 patients were identified as eligible, which included 385 and 1512 patients who received FFX and GnP, respectively. The most common reason for exclusion was the absence of two points of weight data before the initiation of first-line treatment. From the overall population (1897 patients), 421 patients (22.2%) and 1476 patients (77.8%) were incorporated into the cachexia and non-cachexia groups, respectively. Of the 385 patients who received FFX, 94 patients (24.4%) were identified as cachexia and 291 patients (75.6%) were not. Of the 1512 patients who received GnP, 327 patients (21.6%) were identified as cachexia and 1185 patients (78.4%) were not.

### Patient characteristics

Baseline characteristics at the initiation of first-line chemotherapy are shown in Table 1. The mean age was 68.7 and 67.7 years in the cachexia and non-cachexia groups, respectively. Patients in the cachexia group had a lower mean baseline weight as compared to patients in the non-cachexia group (54.4 kg vs. 56.5 kg). Patients in both the cachexia group and non-cachexia group were predominately male (59.1% and 56.6%, respectively) with stage IV (74.8% and 76.6%, respectively). In the cachexia group, 155 patients (36.8%) had biliary drainage, and 29 patients (6.9%) had peritoneal dissemination. Patient characteristics by regimen (Additional file 1: Table S1) showed that patients who received GnP were older than those who received FFX regardless of the presence of cachexia, and those in the cachexia group tended to be slightly older and had lower weight as compared to those in the non-cachexia group for both regimens.

**Table 1:** Baseline characteristics in patients with cachexia or without cachexia

	Cachexia (N = 421)	Non-cachexia (N = 1476)
Age, y		
Mean (SD)	68.7 (8.9)	67.7 (9.2)
Range	29–91	33–87
Male, n (%)	249 (59.1)	835 (56.6)
Baseline body weight (kg)		
Mean (SD)	54.4 (11.5)	56.5 (10.9)
Range	31.1–125.1	32.0–107.6
Stage, n (%)		
III	106 (25.2)	345 (23.4)
IV	315 (74.8)	1131 (76.6)
Activities of daily living		
Mean (SD)	98.5 (7.1)	98.5 (8.8)
Range	45–100	0–100
Charlson comorbidity index		
Mean (SD)	6.1 (3.4)	5.8 (3.3)
Range	2–17	2–18
Biliary drainage, yes, n (%)	155 (36.8)	319 (21.6)
Peritoneal dissemination, yes, n (%)	29 (6.9)	71 (4.8)
Abdominal dropsy, yes, n (%)	3 (0.7)	11 (0.7)
C-reactive protein (mg/dL), n (%)		
> 0.5	14 (3.3)	59 (4.0)
≤ 0.5	19 (4.5)	75 (5.1)
Missing	388 (92.2)	1342 (90.9)
Hemoglobin (g/dL), n (%)		
≥ 12	20 (4.8)	81 (5.5)
< 12	13 (3.1)	55 (3.7)
Missing	388 (92.2)	1340 (90.8)
Albumin (g/dL), n (%)		
≥ 3.2	28 (6.7)	116 (7.9)
< 3.2	5 (1.2)	20 (1.4)
Missing	388 (92.2)	1340 (90.8)

SD, standard deviation

### Time-to-treatment failure

**Fig.3:**
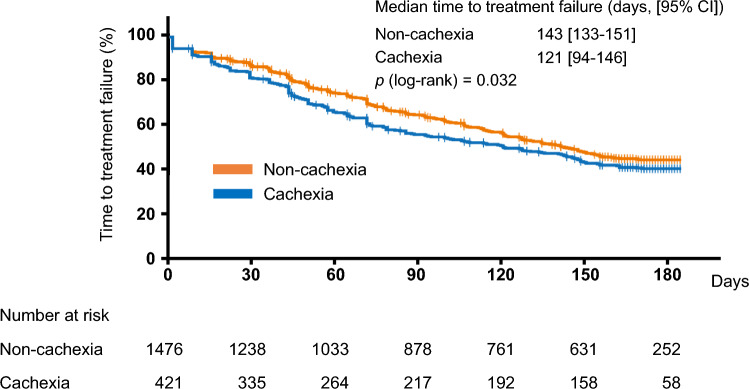
Kaplan–Meier curves of time-to-treatment failure. CI, confidence interval

The median TTF was 121 days (95% CI 94–146) in the cachexia group and 143 days (95% CI 134–152) in the non-cachexia group (Figure 3), with a significant difference (log-rank test; *P*=0.032). The HR for TTF from the Cox regression analysis with the cachexia group versus the non-cachexia group was 1.136 (95% CI 0.979–1.319) (Table 2). TTF by regimen showed that in the 385 patients who received FFX, the median TTF was 64 days (95% CI 55–105) in the cachexia group and 131 days (95% CI, 96–150) in the non-cachexia group (Additional file 1: Figure S1), with a significant difference (log-rank test; *P*=0.017). On the other hand, in the 1512 patients who received GnP, the median TTF was 142 days (95% CI 113–162) in the cachexia group and 147 days (95% CI 113–162) in the non-cachexia group (log-rank test; *P*=0.298; Additional file 1: Figure S2).

**Table 2:** Cox hazard regression for time-to-treatment failure

Confounding factors	Hazard ratio	95% CI
Cachexia or non-cachexia	1.136	0.979–1.319
Age	1.005	0.998–1.013
Sex	0.977	0.843–1.132
Charlson comorbidity index	1.023	1.002–1.044
Baseline body weight	0.998	0.991–1.005
FFX or GnP	1.321	1.127–1.549
Stage	1.252	1.063–1.473
Activities of daily living	0.996	0.989–1.002
Binary drainage	1.049	0.907–1.214
Peritoneal dissemination	0.949	0.714–1.261
Abdominal dropsy	1.168	0.575–2.373

FFX, FOLFIRINOX (fluorouracil + leucovorin + irinotecan + oxaliplatin); GnP, gemcitabine + nab-paclitaxel; CI, confidence interval

### Overall survival

The median OS was not reached in either the cachexia group or non-cachexia group during the follow-up period (six months), with no difference between the two groups (log-rank test; *P*=0.186; Supplementary Figure 3).

### Number of doses

In the patients who received FFX, the median number of doses was five (range, 1–14) in the cachexia group and seven (range, 1–14) in the non-cachexia group (Table 3). Of the patients who received GnP, the median number of doses was nine (range, 1–20) in the cachexia group and 11 (range, 1–21) in the non-cachexia group. Results from generalized linear model analysis suggested that cachexia was independently associated with a reduced number of doses (beta coefficient, 0.895; 95% CI, 0.819–0.977; *P*=0.014; Additional file 1: Table S2).

**Table 3:** Number of doses by regimen

	FFX		GnP	
	Cachexia N = 94	Non-cachexia (N = 291)	Cachexia (N = 327)	Non-cachexia (N = 1185)
Number of doses, median (range)	5 (1–14)	7 (1–14)	9 (1–20)	11 (1–21)

FFX, FOLFIRINOX (fluorouracil + leucovorin + irinotecan + oxaliplatin); GnP, gemcitabine + nab-paclitaxel

### Relative dose intensity

RDI for each regimen component is summarized in Supplementary Table 3. No significant difference in the mean RDI of each component of the FFX regimen was observed between the cachexia group and the non-cachexia group. The mean RDI of gemcitabine and nab-paclitaxel in the cachexia group was 0.674 and 0.658, respectively, and that in the non-cachexia group was 0.701 and 0.696, respectively, with a significant difference between two groups.

### Changes in body weight from the initiation of first-line chemotherapy

Changes in body weight from the initiation of first-line chemotherapy through 6 months later are shown in Additional file 1: Table S4. The mean changes in body weight from baseline through 6 months were decreased in both cachexia and non-cachexia groups.

## Discussion

This is a retrospective observational cohort study using the MDV database to examine the association between cachexia and the treatment course of first-line chemotherapy with FFX or GnP in Japanese patients with pancreatic cancer. The results suggested that cachexia is associated with shorter TTF and a reduced number of doses.

Of the patients with pancreatic cancer analyzed in this study, 22.2% had cachexia at the initiation of first-line chemotherapy (Figure 2). A previous single-center, retrospective, observational study in Japan showed higher incidence of cachexia (50%) in patients with pancreatic cancer at the initiation of first-line chemotherapy [18]. One possible reason for the difference was the different diagnostic criteria used in the two studies; the previous study included weight loss of > 2% with a body mass index (BMI) < 20 kg/m^2^, whereas the current study used weight loss of ≥ 5%.

The median TTF was shorter in the cachexia group than in the non-cachexia group (Figure 3). The negative effect of cachexia on TTF in initial chemotherapy was also suggested in the previous observational study [14]. Similar trends have also been reported in other cancer types, such as lung cancer, gastric cancer, and head and neck cancer [20–22]. In the TTF analysis by regimen, TTF for FFX treatment was significantly shorter in the cachexia group than in the non-cachexia group; however, no such difference was observed in TTF for GnP treatment (Additional file 1: Figures S1 and S2), suggesting that the FFX regimen was more likely to be affected by cachexia. Of note, the median TTF for FFX treatment in the cachexia group was only 64 days. Because modified FFX was associated with higher incidences of severe anorexia and diarrhea compared to GnP in the real-world setting [23], patients receiving FFX in the cachexia group might have had a higher treatment discontinuation rate. Thus, GnP regimen rather than FFX regimen may be suggested for patients with cancer cachexia to prolong their TTF.

We examined the number of doses, RDI, and OS as exploratory outcomes. Regarding the number of doses, we found that for both FFX and GnP treatment, patients in the cachexia group had two fewer doses compared to those without cachexia, suggesting that cachexia might be associated with the reduced number of doses of both regimens. In the patients receiving GnP, the difference between the cachexia group and the non-cachexia group was observed only in the number of doses and not in the TTF. While TTF represents the entire duration of treatment, which may include a period of temporary interruption, the number of doses represents the number of prescriptions during the TTF period; thus, the number of doses would reflect any interruption of drugs. The fact that the only the number of doses was reduced in patients receiving GnP may indicate that the frequency of treatment resumption after temporal interruption may have been higher in the patients than those receiving FFX. It should be noted that other confounding factors including age, Charlson comorbidity index, stage, ADL, and regimen type might have affected the number of doses, as indicated by the generalized linear model analysis result (Additional file 1: Table S2). RDI for both gemcitabine and nab-paclitaxel was reduced in patients with cachexia compared to those without cachexia (Additional file 1: Table S3), which was consistent with the previous result [14]. Although statistically significant, the observed difference was marginal. In the current study that had only a 6-month follow-up period, the two groups showed no significant difference in OS (Additional file 1: Figure S3), but further follow-ups and information of death after hospital discharge may be required to detect any difference.

Treatment options for cachexia had been limited before anamorelin, an orally active, highly selective ghrelin receptor agonist [24], was approved in Japan for patients with cachexia associated with non-small cell lung cancer, gastric cancer, colorectal cancer, and pancreatic cancer. Anamorelin demonstrated improvements in lean body mass, body weight, and appetite in patients with cachexia in phase II/III clinical trials [25–28], and it also increased prealbumin, a nutritional state marker, suggesting an improved nutritional status [26, 27]. Thus, treatment of cachexia with anamorelin may have a positive effect on the clinical outcome of chemotherapy in patients with cachexia.

This study has several limitations. First, although the MDV database is one of the largest databases in Japan, it does not cover information from most of academic medical centers. Second, current analysis was based on the information in same hospital, and it did not follow information after hospital transfer. Third, the database includes body weight data but lacks some important clinical information such as laboratory data. Therefore, we were able to use solely body weight (≥5% weight loss in the preceding six months) to define cachexia. Fourth, body weight data are recorded at only hospitalization, and this study only targeted patients who had their body weight recorded at least twice within six months before the initiation of first-line chemotherapy, which may have caused a selection bias. Fifth, because the claims data only recorded the prescription in terms of the number of ampoules but not the exact amount, we could not distinguish between the conventional FFX and modified FFX. Sixth, because the MDV database does not include patients’ body composition data and information regarding reasons for treatment discontinuation, those could not be incorporated in analyses. Lastly, the OS analysis in this study was based solely on the events during hospitalization.

## Conclusion

We examined the association between cancer cachexia and treatment course of first-line chemotherapy (FFX or GnP) in patients with advanced pancreatic cancer in real clinical contexts in Japan. By using real-world clinical data, we found that cachexia tends to be associated with shorter TTF and a reduced number of doses.

### Supplementary Information

Below is the link to the electronic supplementary material.

**Additional file 1: Figure S1.** TTF (Patients received FFX). **Figure S2.** TTF (Patients received GnP). **Figure S3.** OS. **Table S1.** Patient characteristics by regimens. **Table S2**. GLM analysis for number of doses. **Table S3.** RDI. **Table S4.** Changes in body weight from baseline after the initiation of first-line chemotherapy.

## Data Availability

Qualified researchers may request Ono Pharmaceutical Co., Ltd. to disclose individual patient-level data from clinical studies through the following website: https://www.clinicalstudydatarequest.com/. For more information on Ono Pharmaceutical Co., Ltd.’s Policy for the Disclosure of Clinical Study Data, please see the following website: https://www.ono-pharma.com/en/company/policies/clinical_trial_data_transparency_policy.html

## Abbreviations

ADL: Activities of daily living
CI: Confidence interval
FFX: FOLFIRINOX
GnP: Gemcitabine plus nab-paclitaxel
HR: Hazard ratio
ICD-10: International Statistical Classification of Diseases and Related Health Problems 10th edition
MDV: Medical Data Vision
OS: Overall survival
RDI: Relative dose intensity
TTF: Time-to-treatment failure
