# Supplementary material for: Effect of cancer cachexia on first-line chemotherapy in patients with advanced pancreatic cancer: a claims database study in Japan
Source: Int J Clin Oncol. 2024 Feb 14;29(4):456–63. doi: 10.1007/s10147-024-02467-6 (PMC10963515; doi:10.1007/s10147-024-02467-6)
Supplement: Supplementary file 1 — Additional file 1: Figure S1. TTF (Patients received FFX). Figure S2. TTF (Patients received GnP). Figure S3. OS. Table S1. Patient characteristics by regimens. Table S2. GLM analysis for number of doses. Table S3. RDI. Table S4. Changes in body weight from baseline after the initiation of first-line chemotherapy. [file 10147_2024_2467_MOESM1_ESM.pdf]

Supporting Information

Effect of cancer cachexia on first-line chemotherapy in patients with advanced pancreatic cancer: a claims database study in Japan

Junji Furuse, Fumihiko Osugi, Koji Machii, Koji Niibe, Toshimitsu Endo

- Figures
  - Supplementary Figure 1. TTF (Patients received FFX)
  - Supplementary Figure 2. TTF (Patients received GnP)
  - Supplementary Figure 3. OS
- Tables
  - Supplementary Table 1. Patient characteristics by regimens
  - Supplementary Table 2. GLM analysis for number of doses
  - Supplementary Table 3. RDI
  - Supplementary Table 4. Changes in body weight from baseline after the initiation of first-line chemotherapy

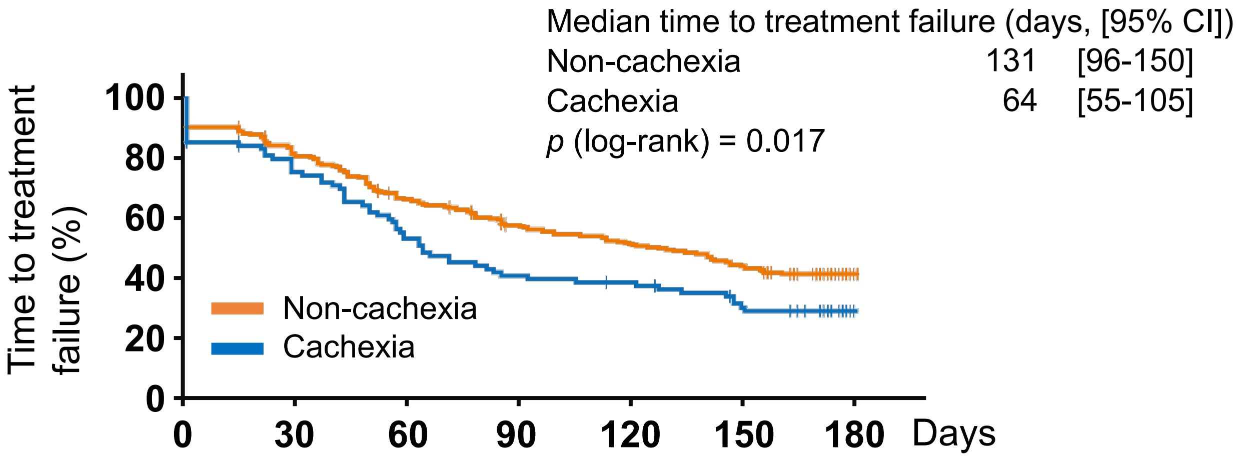

Number at risk							
Non-cachexia	291	232	188	160	143	123	40
Cachexia	94	69	49	38	35	26	7

Supplementary Figure 1 Kaplan–Meier curves of time to treatment failure in the patients who received FOLFIRINOX. CI, confidence interval

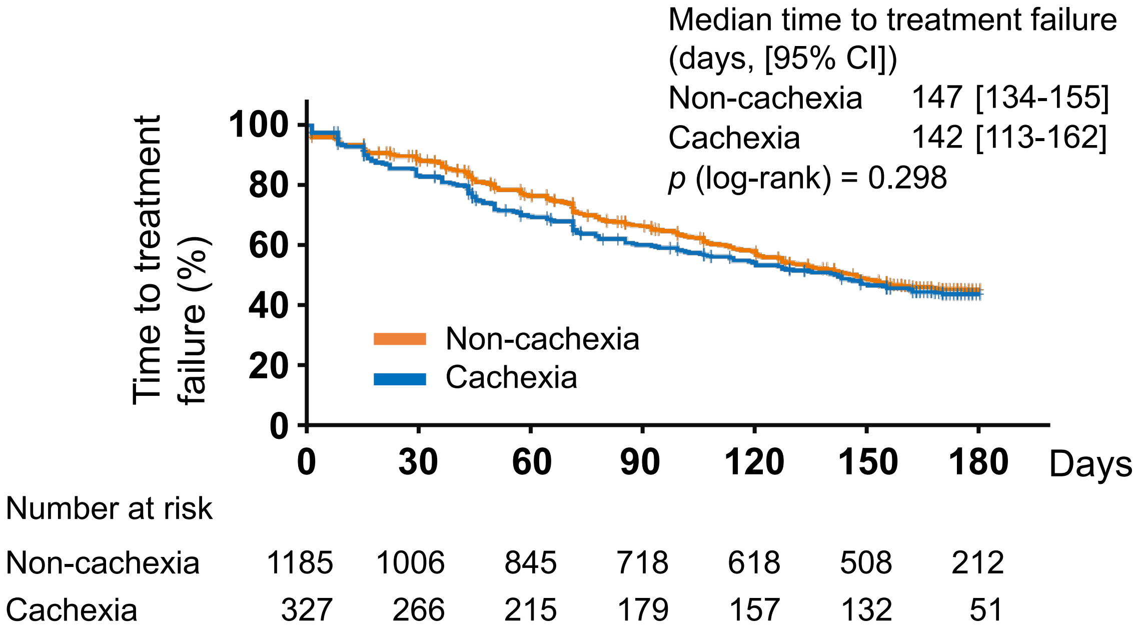

Supplementary Figure 2. Kaplan–Meier curves of time to treatment failure in the patients who received gemcitabine + nab-paclitaxel. CI, confidence interval

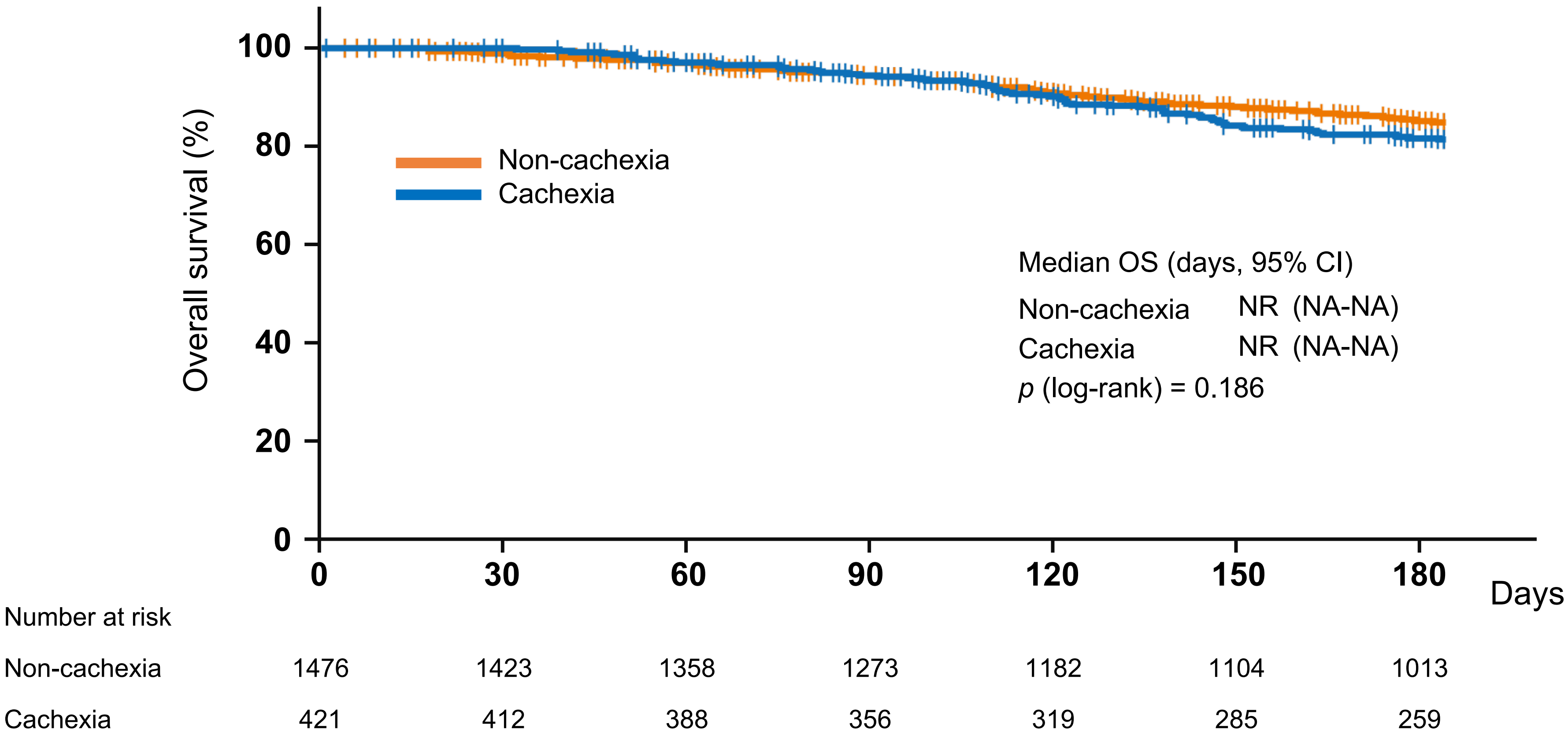

Supplementary Figure 3. Kaplan–Meier curves of overall survival. CI, confidence interval; NR, not reached; NA, not applicable.

Supplementary Table 1. Patient characteristics by regimens.

	FFX		GnP	
	**Cachexia n = 94**	**Non-cachexia n = 291**	**Cachexia n = 327**	**Non-cachexia n = 1,185**
Age, y				
Mean	63.5	61.6	70.2	69.2
SD (range)	8.3 (38–78)	9.1 (33–87)	8.5 (29–91)	8.6 (36–87)
Sex, male (%)	55 (58.5)	165 (56.7)	194 (59.3)	670 (56.5)
Baseline body weight, kg				
Mean	57.1	58.6	53.7	56.0
SD (range)	11.2 (31.9–83.9)	11.9 (34.5–107.6)	11.4 (31.1–125.1)	10.6 (32.0–97.0)
Stage, n (%)				
III	25 (26.6)	65 (22.3)	81 (24.8)	280 (23.6)
IV	69 (73.4)	226 (77.7)	246 (75.2)	905 (76.4)
Activities of daily living				
Mean	99.7	99.8	98.1	98.1
SD (range)	1.9 (85–100)	1.9 (70–100)	8.0 (45–100)	9.8 (0–100)
Charlson comorbidity index				
Mean	6.1	5.8	6.1	5.8
SD (range)	3.2 (2–13)	3.2 (2–13)	3.4 (2–17)	3.3 (2–18)
Biliary drainage, yes, n (%)	29 (30.9)	49 (16.8)	126 (38.5)	270 (22.8)
Peritoneal dissemination, yes, n (%)	5 (5.3)	16 (5.5)	24 (7.3)	55 (4.6)
Abdominal dropsy, yes, n (%)	0 (0.0)	1 (0.3)	3 (0.9)	10 (0.8)

FFX, FOLFIRINOX (fluorouracil + leucovorin + irinotecan + oxaliplatin); GnP, gemcitabine + nab-paclitaxel; SD, standard deviation

Supplementary Table 2. Generalized linear model analysis.

**Confounding factors**	**Beta coefficient**	**95% CI**	***p*-value**
Cachexia or Non-cachexia	0.895	0.819–0.977	0.014
Age	0.992	0.988–0.996	<0.01
Sex	0.987	0.907–1.074	0.762
Charlson comorbidity index	0.981	0.970–0.993	<0.01
Baseline body weight	1.001	0.997–1.005	0.652
FFX or GnP	0.579	0.526–0.637	<0.01
Stage	0.905	0.826–0.991	0.032
Activities of daily living	1.006	1.002–1.011	<0.01
Biliary drainage	0.939	0.862–1.023	0.152
peritoneal dissemination	0.991	0.839–1.171	0.917
abdominal dropsy	0.918	0.598–1.409	0.696
Intercept	12.607	6.196–25.651	<0.01

CI, confidence interval; FFX FOLFIRINOX (fluorouracil + leucovorin + irinotecan + oxaliplatin;
GnP, gemcitabine + nab-paclitaxel

Supplementary Table 3. Relative dose intensity.

	Cachexia		Non-cachexia		Difference (95% CI)	*p*-value
	**n**	**RDI Mean**	**n**	**RDI Mean**		
Irinotecan	94	0.705	290	0.688	-0.017 (-0.056, 0.022)	0.398
Leucovorin	94	0.810	290	0.792	-0.018 (-0.057, 0.021)	0.359
Oxaliplatin	94	0.843	290	0.795	-0.048 (-0.100, 0.004)	0.069
Fluorouracil	94	0.818	290	0.800	-0.018 (-0.058, 0.022)	0.371
Gemcitabine	327	0.674	1,185	0.701	0.028 (0.0029, 0.052)	0.029
Nab-paclitaxel	327	0.658	1,185	0.696	0.038 (0.0091, 0.067)	0.010

CI, confidence interval; RDI, relative dose intensity

Supplementary Table 4. Percent changes in body weight from the initiation of first-line chemotherapy

	**Cachexia**		**Non-cachexia**	
	**n**	Mean (SD)	**n**	Mean (SD)
Day 1 to 30	154	-0.71 (5.71)	465	-1.37 (4.14)
Day 31 to 60	106	-1.50 (6.77)	286	-4.12 (6.33)
Day 61 to 90	109	-4.12 (8.55)	288	-4.17 (6.96)
Day 91 to 120	87	-3.68 (8.30)	252	-5.04 (7.93)
Day 121 to 150	76	-3.98 (10.96)	249	-4.26 (7.71)
Day 151 to 180	63	-3.41 (10.65)	218	-4.69 (8.12)

SD, standard deviation
